# A case of Pseudomyxoma Peritonei of an unexpected origin

**DOI:** 10.1186/s13000-021-01179-z

**Published:** 2021-12-20

**Authors:** Marie Csanyi-Bastien, France Blanchard, Aude Lamy, Jean-Christophe Sabourin

**Affiliations:** 1 Department of Pathology, Rouen University Hospital, 1 rue de Germont, 76000 Rouen Cedex, France; 2 INSERM 1245, Rouen University, Rouen, France; 3 Department of Pathology, Normandy University, Caen, France

**Keywords:** Pseudomyxoma peritonei, Ovarian pseudomyxoma peritonei, Appendiceal pseudomyxoma peritonei, Ovarian teratoma, *KRAS* / *GNAS* mutations, LAMN

## Abstract

**Background:**

Pseudomyxoma peritonei (PMP) is a complex and partially understood disease defined by mucin deposits in the peritoneal cavity, mostly of appendiceal origin caused by the rupture of a mucocele often containing Low or High grade Appendiceal Mucinous Neoplasm (LAMN/HAMN). Other origins include primitive ovarian mucinous cystadenoma or cystadenocarcinoma almost always with an associated teratoma, but to our knowledge no case of ovarian teratomatous appendiceal-like mucocele with LAMN has been reported as a cause of PMP.

**Case presentation:**

A 25-year old female with infertility was diagnosed with an isolated left ovarian tumor in a context of PMP. Histological examination revealed an ovarian teratoma containing an appendiceal-like structure with mucocele and LAMN, without any associated lesion of the appendix on full histological analysis. Molecular characterization of the ovarian lesion showed co-*KRAS* and *GNAS* mutations, as described in PMP of appendiceal origin, while only *KRAS* mutations are reported in primitive ovarian mucinous tumor.

**Conclusions:**

Detection of co-*KRAS* and *GNAS* mutations in our case of ovarian teratomatous appendiceal-like mucocele with LAMN shows that when PMP derives from a mucinous ovarian lesion (with histological proof of none-appendiceal involvement), it is probably of a digestive teratomatous origin, emphasizing the need to actively search for tetatomatous signs in a context of ovarian PMP.

## Background

Pseudomyxoma Peritonei (PMP) is a rare neoplastic disease defined by the presence of mucinous ascites or mucinous deposits in the peritoneal cavity. It is a rare disease whose incidence is approximately 1 to 2 cases per million people per year and which affects more commonly women. Its clinical manifestations are abdominal distention, pain and transit disorder. Its complex physiopathology remains unclear. The majority of PMP are of appendiceal origin due to rupture of a mucocele associated with a low-grade appendiceal mucinous neoplasm (LAMN) or a high-grade appendiceal mucinous neoplasm (HAMN). However, in a few cases, tumors of other origins, especially ovarian, may be associated with PMP. Indeed, the description of authentic cases of PMP without appendiceal involvement but with associated ovarian tumor have confirmed its ovarian origin. Primitive ovarian PMP are thought to develop from a broad spectrum of mucinous ovarian entities, from mucinous cystadenoma to borderline cystadenoma and cystadenocarcinoma, almost always in the context of an associated teratoma. Mucinous proliferation in teratoma is well described but to our knowledge, no teratomatous LAMN has been reported. We describe a case of PMP caused by a ruptured appendiceal-like mucocele associated with LAMN in an ovarian teratoma. Our investigations provide clinical, histological, immunohistochemical and molecular data. We also conducted a literature review about PMP origin, especially ovarian.

## Case presentation

### Clinical context

A 25-year-old woman, without previous medical history, presented for infertility lasting for more than one year. Clinical examination was normal but abdominal and pelvic computed tomodensitometry (CT) revealed a cyst of the left ovary associated with abundant peritoneal ascites that could correspond to mucinous material. Pelvic magnetic resonance imaging (MRI) confirmed ascites and showed a heterogeneous mass of the left ovary measuring 8.4 × 6.8 cm with adipose, solid and cystic regions that were suggestive of a dermoid cyst. The right ovary and uterus seemed normal. No other lesion was seen in the rest of the body, notably in the digestive system. In this context, surgery by left oophorectomy with appendicectomy and omentectomy was performed 3 months after the first consultation, without resorting to additional hyperthermic intraperitoneal chemotherapy (HIPEC). Intra-operative examination revealed mucinous material inside the peritoneal cavity and a normal digestive tract with a normal appendix. There was no complication of the surgery. The 5-month follow-up based on clinical and imaging surveillance revealed no complaints. Without relapse, the patient was able to pursue her plan to have a child.

### Histopathological findings

**Fig. 1:**
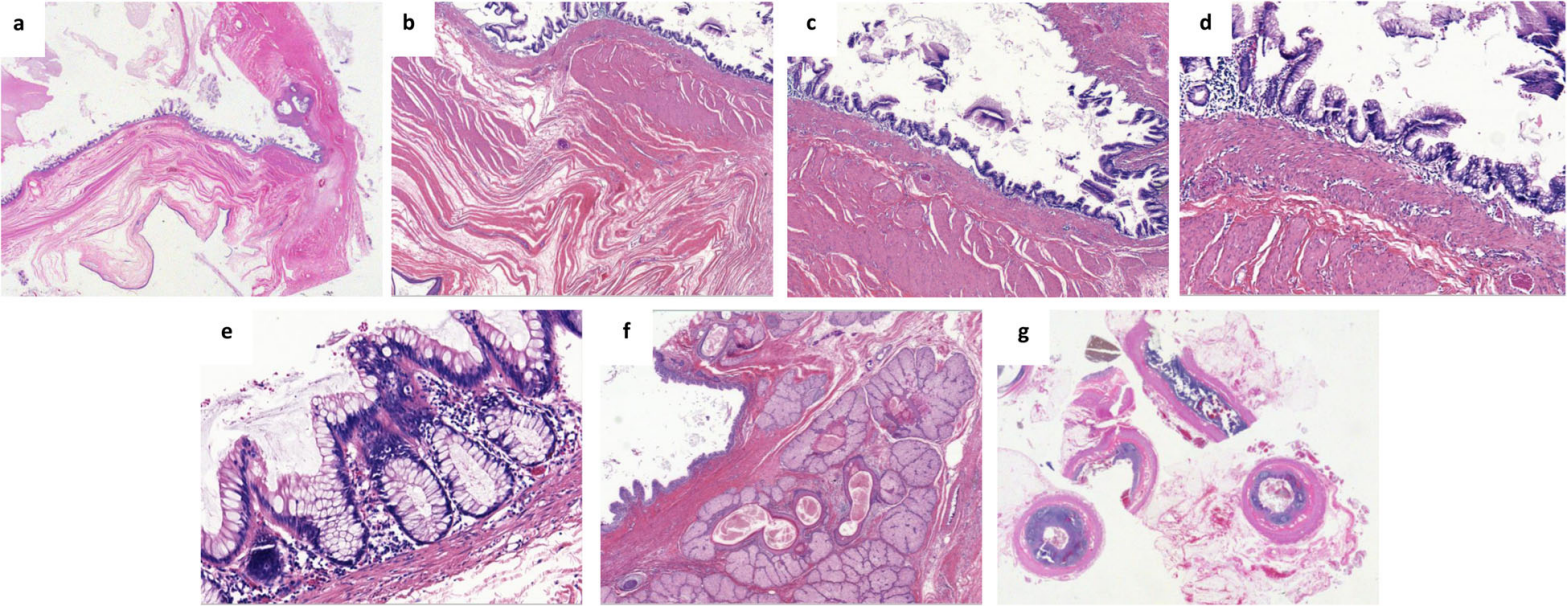
Histological aspects of the ovarian teratoma (a-f) and the normal appendix (g). Views of the organoid colonic/appendiceal wall inside the ovarian teratoma at the top, composed of mucosa, muscularis mucosae, submucosa and muscularis propria, with dissociated mucus at left, close to some squamous teratomatous element at the bottom, without any magnification (a) and with low-power magnification Gx25 (b). Focus on colonic/appendiceal LAMN comprising elongated glands layered by mild atypical proliferating cells producing mucin at magnification Gx50 (c), Gx100 (d) and Gx200 (e). View of other teratomatous elements, mostly of cutaneous epithelium and glands at magnification Gx50 (f), and of the histologically normal appendix without magnification (g)

**Fig. 2:**
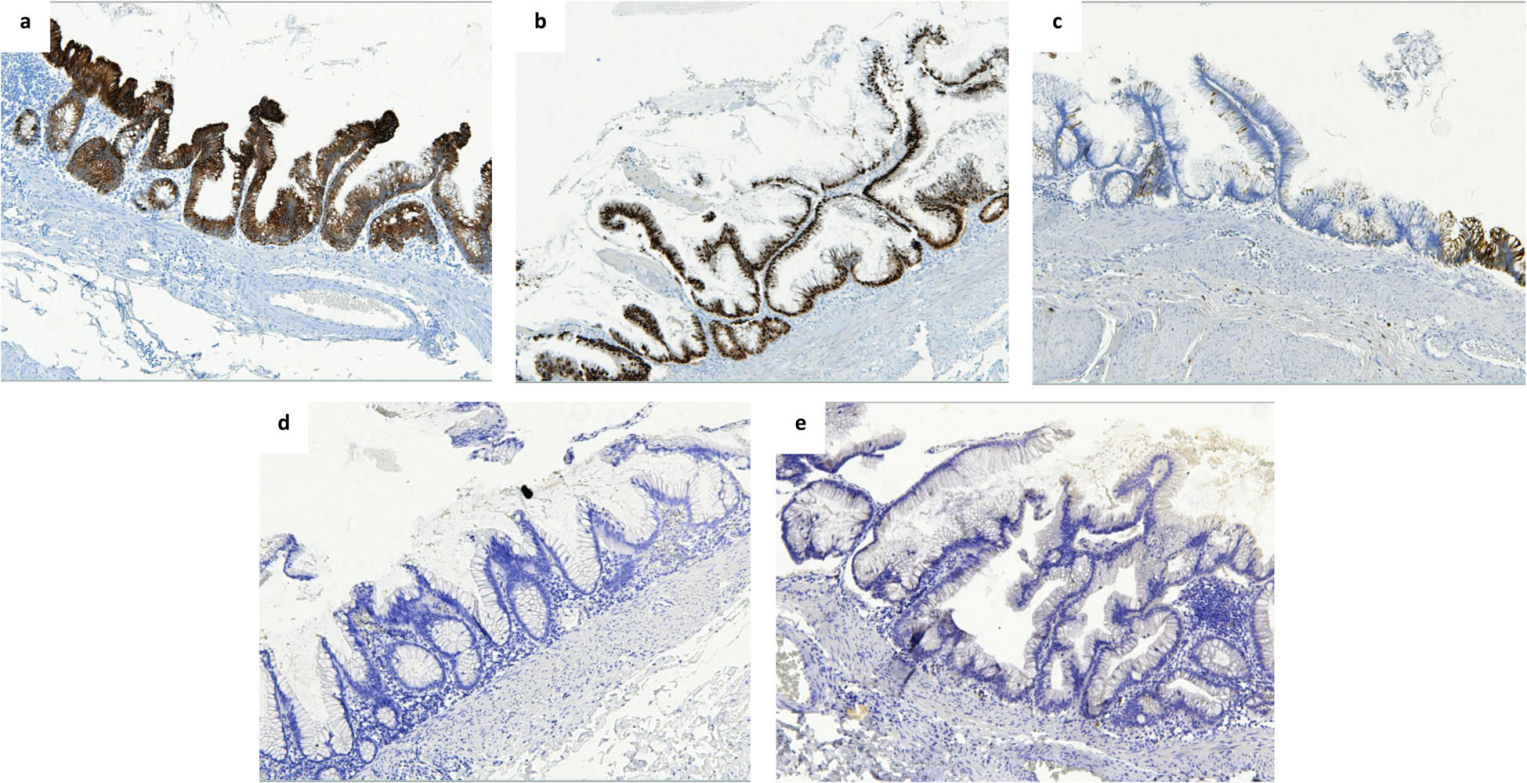
Immunohistochemical profile of the colonic epithelium with LAMN inside the ovarian teratoma. The epithelial cells are strongly stained by CK20 (a) and CDX2 (b) antibodies. They are heterogeneously stained by CK7 (c) antibody. They present no staining by estrogen (d) and progesterone (e) receptors

Macroscopically, the left ovary was cystic measuring 9.5 × 7 × 7 cm and weighing 305 g. It was ruptured on 4 cm. Its cut section revealed a heterogeneous and viscous mass with hair. The appendix, measuring 6 cm in length, and the omentum were macroscopically normal. Histologically, the ovarian cyst corresponded to a mature pluritissular teratoma with intermingled skin and pilosebaceous annexes, serous and mucinous glands, respiratory epithelium, adipose tissue and smooth muscle **(**Fig. 1**)**. The organoid areas with the aspect of a colon, representing about 20% of the ovarian cyst, were composed of colonic mucosa, muscularis mucosae, and submucosa from the surface to the depth. A thick muscularis propria was also observed. In the colonic mucosa, some glands were elongated and layered with moderate proliferating epithelial cells with minimal atypia, near to mucin pools stained with Alcian blue. The colonic epithelial cells were immunohistochemistry stained with CK20 and CDX2, and showed heterogeneous staining for CK7. These cells were negative for estrogen and progesterone receptors **(**Fig. 2**)**. The ovarian surface was covered with hyperplastic mesothelial cells and presented acellular mucinous pools, also found in the omentum. The left fallopian tube was normal. The appendix examined in totality was histologically normal besides mucin deposits on the surface of the serosa. It did not present any mucocele or LAMN/HAMN. All together, these data suggested a diagnosis of acellular PMP (according to Carr classification [1]) caused by a ruptured appendiceal-like mucocele associated with LAMN, in a left ovarian teratoma.

### Molecular features

**Fig. 3:**
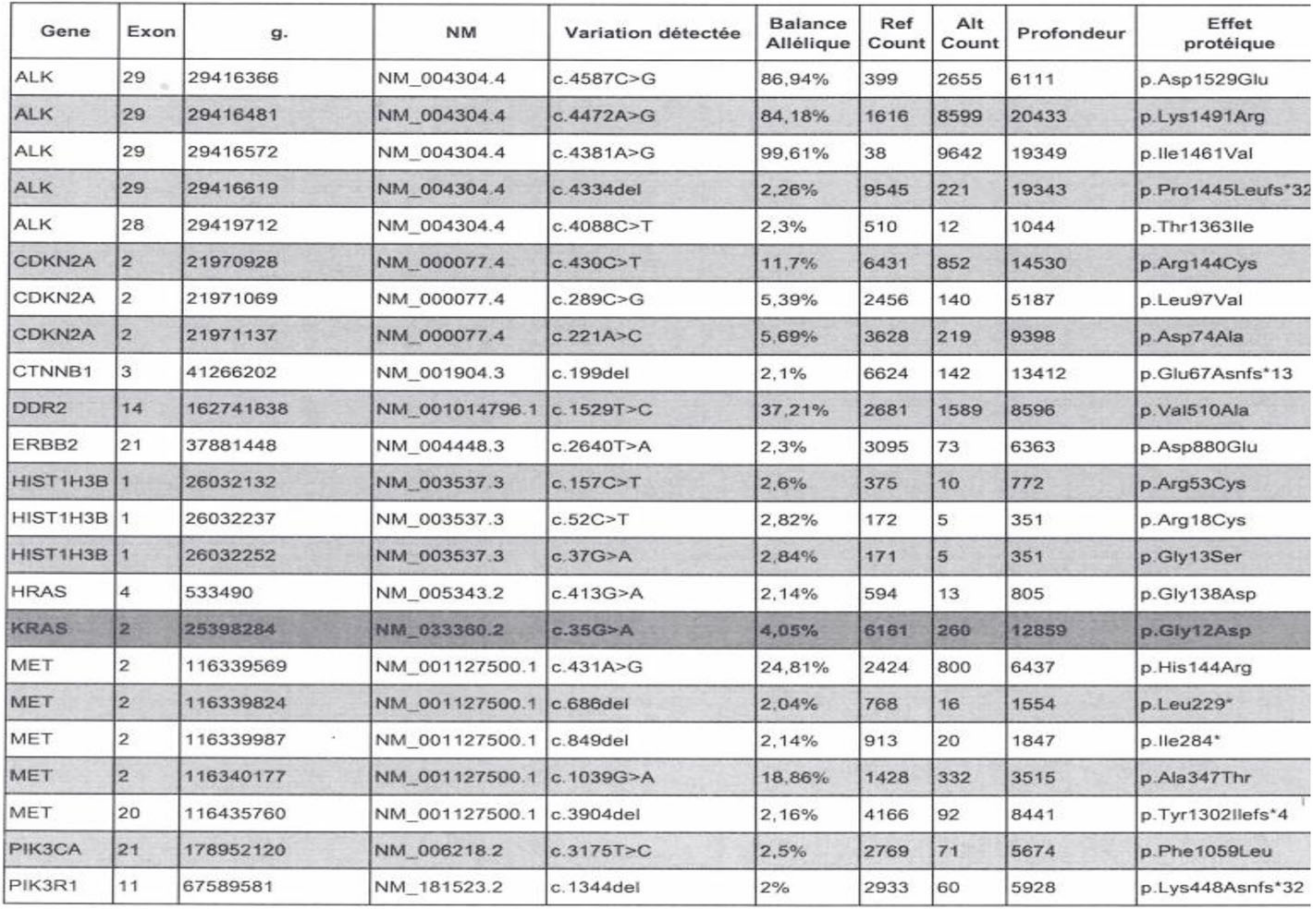
List of mutations found in the teratomatous LAMN. Among the mutations revealed in the LAMN lesion, there was a c.35G > A mutation in *KRAS* gene corresponding to a p.(Gly12Asp) protein effect (highlighted line)

**Fig. 4:**
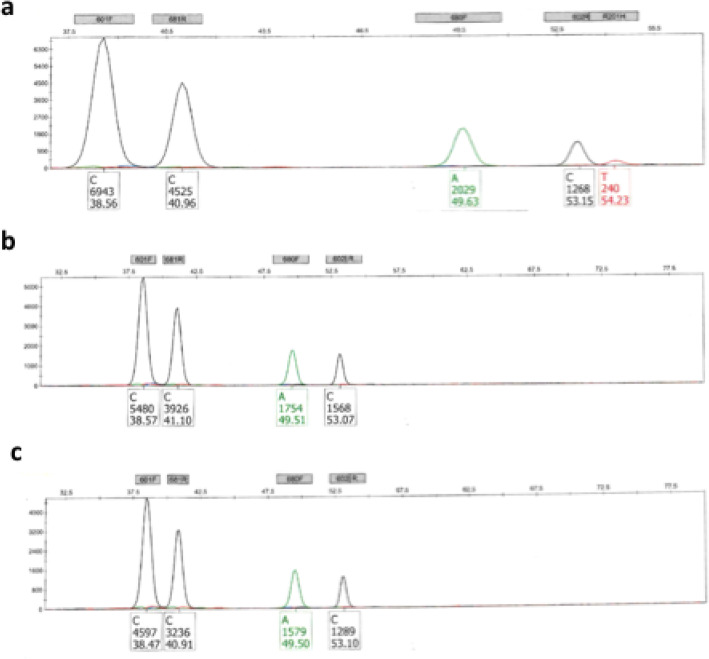
Mutational status of *GNAS* gene in teratomatous LAMN, ovarian teratoma and appendix by SNaPshot analysis. In the teratomatous LAMN there was a c.602C > T (reverse) corresponding to a c.602G > A (forward) mutation in *GNAS* (a) while in the other tissues of the teratoma (b) and in the normal appendix (c) there was no mutation detected in *GNAS*

Next generation sequencing of the LAMN of the teratomatous mucocele revealed an activating mutation of *KRAS* gene c.35G > A corresponding to the p.(Gly12Asp) substitution **(**Fig. 3**)**. Complementary molecular analysis by SNaPshot showed an associated mutation of *GNAS* c.602G > A resulting in p.(Arg201His). No mutation was found by these two techniques on the other tissues of the ovarian teratoma (squamous, respiratory, adipose or smooth muscle elements) or in the normal appendix and ovarian parenchyma **(**Fig. 4**)**.

## Discussion

PMP is caused by the peritoneal localization of mucin, almost exclusively in the context of a tumor, by two possible mechanisms: tumor rupture in the peritoneal cavity causing the spread of mucinous material containing a variable amount of tumor cells, or peritoneal metastasis of a mucinous adenocarcinoma. Most of the tumors (90%) are of appendiceal origin from LAMN or HAMN associated with a mucocele [2]. Other tumors were mucinous adenocarcinomas of colonic [3], gastric [4], pancreatic [5, 6], urachian [7], pulmonary [8], endocervical [9] or mammary [10] origin, or to rare mucinous ovarian tumors, cystadenomas or cystadenocarcinomas [9]. Some authors reported non-neoplastic intra-peritoneal mucinous deposits caused by alternative processes as mucin retention due to a stercolith or a diverticule, or mucinous metaplasia of fallopian tubes [11–13], but such situations are very rare and questionable.

Physiopathologically, the mucin deposits in peritoneal cavity, more or less associated with tumor cells, are caused by the redistribution phenomenon [14] and epithelio-mesenchymal transition [15]. Hematogenic and lymphatic routes of dissemination seem to be infrequent in this complex pathology, still partially understood. However, because of its clinical evolution, PMP is considered as a neoplastic condition with variable behaviors, either indolent or aggressive.

Curative treatment of PMP relies on maximal cytoreduction surgery completed by HIPEC, performed by experienced staff in a reference center [16, 17]. This procedure is indeed associated with numerous potential complications, leading to high morbidity (16 to 65%) and mortality (0 to 18%). Nevertheless, extended survey is possible (59% at 5 years) [18].

Several histological classifications have been established for PMP. In 2017, Carr et al. proposed a PMP classification divided in four categories: mucin without epithelial cells; PMP with low-grade histological features; PMP with high-grade histological features; and PMP with signet ring cells [1]. Recently, the 2019 WHO (World Health Organization) classification adopted a three-tiered system of classification to unify and simplify denomination of the disease. Grade 1 (or low grade appendiceal mucinous neoplasm) is defined by acellular or hypocellular mucinous deposits, with pushing tumor margins, and low grade epithelial cell cytology. Grade 2 (or high grade appendiceal mucinous neoplasm) is characterized by mucinous deposits with numerous epithelial cells often arranged in clusters with marked atypia. Grade 3 (or high grade with signet-ring cells) is represented by neoplasms containing true signet-ring cells defined as intracytoplasmic mucin vacuole indenting the nucleus (degenerating cells floating within mucin pools should not be considered as true signet-ring cells) [19].

The primitive ovarian origin of PMP has been debated for a long time. First descriptions of PMP were from appendiceal or intestinal origins. Thus, when a female patient presented with a clinical situation of PMP with both mucinous lesions of the appendix and the ovaries, she was considered to present either a PMP of appendiceal origin with secondary localization of the ovaries or an appendiceal PMP with a concomitant borderline mucinous ovarian tumor [20–22]. In order to elucidate the origin of PMP in cases of both appendiceal and ovarian mucinous lesions, some authors tried to define morphological criteria comparing the aspects of mucinous tumor of the ovaries with and without PMP and appendiceal tumor.

For Ronett et al., the secondary ovarian localization of a primitive digestive tumor was retained when (i) the ovarian tumor involved the surface with eventually superficial stroma; (ii) ovaries were of a quite normal size; (iii) a unilateral ovarian tumor had a digestive phenotype in a context of anteriority of such a digestive tumor; (iv) a bilateral ovarian tumor had a digestive phenotype without any known antecedent; (v) an appendiceal tumor was ruptured with an intact associated ovarian tumor [22].

Stewart et al. observed that secondary ovarian tumor was made by scalloped glands layered by sub epithelial clefts while primitive ovarian tumor did not share these features but was instead associated with an abundant stroma reaction and histiocytic infiltration [23].

Immunohistochemistry has also been used to distinguish between primary and secondary ovarian origin. Ferraira et al. showed that CK20 and MUC2 were more often expressed by mucinous ovarian tumors associated with PMP than by mucinous ovarian tumors without PMP, supporting the hypothesis of a secondary ovarian localization of a primitive digestive tumor [24]. Nevertheless, Saluja et al. reported a case of PMP associated with an ovarian borderline mucinous tumor without any digestive tumor [24]. In this case, the appendix was normal on full microscopic examination, and the ovarian tumor expressed both CK7 and CK20, with MUC2. Finally, studies revealed that the immunohistochemical profile of the tumor did not allow to distinguish between PMP of primitive ovarian or digestive origin, both of them being positive for CK20 and CDX2 with variable staining for CK7 [25].

O’Connell et al. investigated the mucin composition of PMP, which is principally made of MUC2 and MUC5AC and revealed that while MUC2 was more abundant in PMP, but also in appendiceal mucinous tumors and normal digestive tissue, MUC5AC was predominant in mucinous ovarian primitive tumors, suggesting that mucin composition could help to distinguish the origin of PMP [26, 27]. However, only a few studies on mucin composition are available.

Nonetheless, some cases of authentic PMP with an ovarian origin have been described. These cases were associated with various mucinous tumors of the ovary, as benign mucinous adenomas, borderline mucinous tumors and adenocarcinomas [28–37]. In many of these primitive ovarian PMP, in which an appendiceal origin was formally excluded, mucinous ovarian tumors were associated with teratoma, leading some authors to consider that primitive ovarian origin of a PMP was only possible in a context of a mucinous ovarian tumor arising from an ovarian teratoma [36]. It should be noted that ovarian teratomas associated with mucinous tumors causing PMP did not show particularities from other teratomas without mucinous associated lesion, but such teratomatous component was often a minor part of the lesion. All the reported cases of primitive ovarian PMP associated with a teratoma in literature, from 2003 till 2021, have been listed in Table 1.

**Table 1:** List of reported cases in literature of primitive ovarian PMP associated with teratoma

Number of reported cases	Age at diagnosis	Laterality / size of ovarian tumor	Histological type of mucinous ovarian tumor	Immunohistochemical profile of mucinous ovarian tumor	Percentage and composition of teratomatous elements	Appendix examination	Therapeutic strategy	Follow-up data	Bibliographical reference
3	35	right / 22 cm	borderline with focal cystadenocarcinoma	CK7-, CK20+	4%, squamous epithelium and adnexal glands	Ma and Mi: normal	cytoreductive surgery	death after disease progresion at 49 mo	[28] Ronnett et al. Am J Surg Pathol. 2003.
	81	left / 7 cm	cystadenoma	CK7-, CK20+	8%, squamous epithelium and sebaceous glands	Ma and Mi: normal	cytoreductive surgery	survival without relapse at 54 mo	
	89	left / 15 cm	borderline	CK7-, CK20+	11%, squamous epithelium and cartilage	Ma and Mi: normal	cytoreductive surgery	survival without relapse at 48 mo	
1	39	left / 14 cm	cystadenoma	CK7-, CK20+	low, squamous epithelium and adnexal glands	Ma and Mi: normal	cytoreductive surgery	survival without relapse at 6 mo	[29] Pranesh et al. J Clin Pathol. 2005.
1	67	left / 20 cm	borderline	CK7-, CK20+	low, dermoid cyst	NA (appendicectomy 42 years ago)	cytoreductive surgery	survival without relapse at 6 mo	[30] Marquette et al. Int J Gynecol Pathol. 2006.
2	38	left / 14 cm	borderline	CK7-, CK20+	5–10%, squamous epithelium and adnexal glands	Ma and Mi: normal	cytoreductive surgery	survival without relapse at 24 mo	[31] Stewart et al. Pathology. 2006.
	58	right / 16.5 cm	borderline	CK7-, CK20+	5–10%, squamous epithelium and adnexal glands	Ma and Mi: normal	cytoreductive surgery	survival without relapse at 21 mo	
1	45	left / 12 cm	cystadenocarcinoma	NA	low, squamous epithelial and sebaceous glands	Ma and Mi: normal	cytoreductive surgery	NA	[32] Mandal et al. Arch Gynecol Obstet. 2008.
10	57	NA / NA	cystadenoma	CK7-, CK20+ (88%) CK7+, CK20- (13%)	low, squamous epithelium and adnexal glands, more or less other mature tissues	Ma and Mi: normal	cytoreductive surgery	NA	[33] McKenney et al. Am J Surg Pathol. 2008.
	36	NA / 25 cm	borderline			Ma and Mi: normal		NA	
	54	NA / 14 cm	borderline			Ma and Mi: normal		survival without relapse at 24 mo	
	41	NA / 25 cm	borderline			NA		NA	
	42	NA / 7 cm	borderline			Ma and Mi: normal		survival without relapse at 27 mo	
	47	NA / 20 cm	borderline			Ma and Mi: normal		survival without relapse at 23 mo	
	28	NA / 22 cm	borderline			Ma and Mi: normal		survival without relapse at 61 mo	
	48	NA / NA	cystadenocarcinoma			NA		metastases to omentum and pelvic serosa at 29 mo	
	21	NA / NA	cystadenocarcinoma			NA		died of other disease at 6 mo	
	56	NA / NA	cystadenocarcinoma			Ma and Mi: normal		metastases to omentum, small bowel serosa and mesentery at diagnosis	
2	46	right / 15 cm	borderline	CK7+, CK20+	NA, squamous epithelium and bone	Ma and Mi: normal	cytoreductive surgery	NA	[34] Hwang et al. Int J Gynecol Pathol. 2009.
	28	left / 40 cm	cystadenoma focally borderline		NA, squamous epithelium and adnexa	Ma without Mi: normal	cytoreductive surgery and adjuvant chemotherapy	NA	
1	43	right / NA	borderline	CK7+, CK20-	NA, squamous epithelium	Ma and Mi: chronic appendicitis	cytoreductive surgery	survival without relapse at 6 mo	[35] Mohtaram et al. Pan Afr Med J. 2013.
1	45	right / 29 cm	borderline	CK7+, CK20-	skin and adnexa	Ma and Mi: normal	cytoreductive surgery	NA	[36] Choi et al. Pathology. 2016.
3	75	left / NA	borderline	CK7+, CK20+	NA	Ma and Mi: appendicitis	cytoreductive surgery	survival without relapse at 25 mo	[37] Yan et al. Cancer Manag Res. 2020.
	45	right / NA	borderline with focal cystadenocarcinoma	CK7+, CK20+	NA	Ma and Mi: appendicitis	cytoreductive surgery and HIPEC	survival with disease at 30 mo	
	53	bilateral / NA	cystadenocarcinoma	CK7-, CK20+	NA	Ma and Mi: appendicitis	cytoreductive surgery and HIPEC	survival with disease at 29 mo	
1	25	left / 8.4 cm	ruptured appendiceal-like mucocele associated with LAMN in an ovarian teratoma	CK7+, CK20+	skin and pilosebaceous annexes, serous and mucinous glands, respiratory epithelium, adipose tissue and smooth muscle and colonic wall	Ma and Mi: normal	cytoreductive surgery	survival without relapse at 5 mo	Present case report
total: 26 cases	mean age at diagnosis: 47.8								

*Ma* macroscopic, *Mi* microscopic, *mo* months, *NA* Not Available, *HIPEC* Hyperthermic IntraPEritoneal Chemotherapy

Cytoreductive surgery relying on hysterectomy, bilateral salpingo-oophorectomy, appendicectomy and omentectomy

The unique and original feature of our case is that the primitive ovarian tumor responsible for the PMP was not a classic mucinous tumor of the ovary associated with a teratoma but a teratomatous appendiceal-like mucocele with LAMN. To our knowledge, such teratomatous involvement has never been described.

Molecular sequencing of PMP revealed frequent *KRAS* and *GNAS* mutations as in mucinous tumors of the appendix. These mutations are frequent in LAMN and HAMN and slightly rarer in mucinous appendiceal adenocarcinomas, which harbor frequent *TP53* mutations as in HAMN but not LAMN [38]. *KRAS* mutations occur in exon 2. *GNAS* mutations are located at codon 201 in c.601 or c.602. Mutations in codon 601 are frequently c.601C > T, resulting in p.(R201C), and those in codon 602 are often c.602G > A resulting in p.(R201H) [39]. While the former is more common in LAMN, the latter is more common in HAMN. Molecular data on primitive mucinous ovarian carcinomas without the context of PMP showed frequent mutations in *KRAS,* without *GNAS* mutation [39]. Choi et al. studied molecular alterations in primitive ovarian mucinous tumor associated with teratoma and PMP. They revealed *KRAS* and *GNAS* associated mutations [36]. These results, as ours, could indicate that ovarian mucinous tumors associated with teratomas and responsible for PMP are in fact of a teratomatous digestive origin. Molecular data on PMP of other origins than appendiceal and ovarian are not available, because of their rarity.

## Conclusions

PMP is a rare neoplastic disease, deriving in most cases from a mucinous appendiceal tumor from LAMN, HAMN or adenocarcinoma, all associated with co-*KRAS* and *GNAS* mutations. Mucinous tumors of other origins, mostly adenocarcinomas, can also cause PMP, among them mucinous ovarian tumors, almost always in a context of concomitant ovarian teratoma. In the literature, molecular data show that *KRAS* and *GNAS* co-mutations are also present in primitive ovarian PMP associated with teratoma.

By reporting here the presence of *KRAS* and *GNAS* mutations in this extremely rare case of primitive ovarian PMP derived from the rupture of a teratomatous appendiceal-like mucocele with LAMN arising in an ovarian teratoma, our results suggest the teratomatous digestive origin of the mucinous ovarian tumors causing PMP. This finding emphasizes the need to actively search for teratomatous signs in a context of primitive ovarian PMP.

## Data Availability

Concerning clinical and sample collection, approval was obtained according to the agreement of the tumor biobank of Rouen University Hospital (tissue sample collection n° DC2008–689).

## Abbreviations

CT: Computed Tomodensitometry
HAMN: High-grade appendiceal mucinous neoplasm
HIPEC: Hyperthermic IntraPEritoneal chemotherapy
LAMN: Low-grade appendiceal mucinous neoplasm
MRI: Magnetic resonance imaging
PMP: PseudoMyxoma Peritonei
WHO: World Health Organization
